# Evidence of Adult Features and Functions of Hepatocytes Differentiated from Human Induced Pluripotent Stem Cells and Self-Organized as Organoids

**DOI:** 10.3390/cells11030537

**Published:** 2022-02-04

**Authors:** Antonietta Messina, Eléanor Luce, Nassima Benzoubir, Mattia Pasqua, Ulysse Pereira, Lydie Humbert, Thibaut Eguether, Dominique Rainteau, Jean-Charles Duclos-Vallée, Cécile Legallais, Anne Dubart-Kupperschmitt

**Affiliations:** 1 UMR_S 1193, INSERM/Université Paris-Saclay, F-94800 Villejuif, France; eleanor.luce@inserm.fr (E.L.); nassima.benzoubir@inserm.fr (N.B.); jean-charles.duclos-vallee@ifbf-institute.org (J.-C.D.-V.); 2 Centre Hépatobiliaire, Fédération Hospitalo-Universitaire (FHU) Hépatinov, AP-HP, Hôpital Paul Brousse, F-94800 Villejuif, France; mpasqua@fondazionerimed.com (M.P.); ulysse.pereira@utc.fr (U.P.); cecile.legallais@utc.fr (C.L.); 3 UMR CNRS 7338 Biomechanics & Bioengineering, Université de Technologie de Compiègne, Sorbonne Universités, 60203 Compiegne, France; 4 Centre de Recherche Saint-Antoine, Sorbonne Université, INSERM, CRSA, AP-HP, Hôpital Saint Antoine, Metomics, 75012 Paris, France; lydie.humbert@sorbonne-universite.fr (L.H.); thibaut.eguether@sorbonne-universite.fr (T.E.); dominique.rainteau@upmc.fr (D.R.)

**Keywords:** liver organoids, hiPSCs, hiPSC-derived hepatocytes, HLCs, self-assembling, hepatic mature functions, bile acids, bile canaliculi network, metabolism, detoxification

## Abstract

Background: Human-induced pluripotent stem cell-derived hepatocytes (iHeps) have been shown to have considerable potential in liver diseases, toxicity, and pharmacological studies. However, there is a growing need to obtain iHeps that are truly similar to primary adult hepatocytes in terms of morphological features and functions. We generated such human iHeps, self-assembled as organoids (iHep-Orgs). Methods: iPSC-derived hepatoblasts were self-assembled into spheroids and differentiated into mature hepatocytes modulating final step of differentiation. Results: In about four weeks of culture, the albumin secretion levels and the complete disappearance of α-fetoprotein from iHep-Orgs suggested the acquisition of a greater degree of maturation than those previously reported. The expression of apical transporters and bile acid secretion evidenced the acquisition of complex hepatocyte polarity as well as the development of a functional and well-defined bile canalicular network confirmed by computational analysis. Activities recorded for CYP450, UGT1A1, and alcohol dehydrogenase, response to hormonal stimulation, and glucose metabolism were also remarkable. Finally, iHep-Orgs displayed a considerable ability to detoxify pathological concentrations of lactate and ammonia. Conclusions: With features similar to those of primary adult hepatocytes, the iHep-Orgs thus produced could be considered as a valuable tool for the development and optimization of preclinical and clinical applications.

## 1. Introduction

Through guided differentiation, human-induced pluripotent stem cells (hiPSCs) offer a reliable source of hepatocytes that are certainly proving themselves useful in numerous tissue engineering and personalized medicine applications [1,2,3,4,5,6]. However, concerns persist regarding the lack of functional maturity and long-term maintenance in culture of hiPSC-derived hepatocytes (iHeps). Indeed, iHeps still display a fetal hepatic phenotype rather than an adult one [7,8] and, in particular, they continue to express fetal markers such as α-fetoprotein (AFP), as well as being poor at reproducing key mature functions, such as the activity of many detoxification enzymes [8,9]. Improved protocols and 3D culture systems have shown that the hepatic functions of iHeps can be enhanced, thus contributing to the study of liver development, diseases and the efficacy of medicinal products [3,4,5,10,11], but hepatocytes with adult features have still not been generated. This hurdle currently remains the principal drawback of iHeps in terms of applications where a considerable degree of maturity is required, such as pharmacology/toxicology studies and the development of in vitro systems to model liver physiology and pathophysiology. Furthermore, the potential for iHeps to offer an unlimited source of cells makes them an attractive tool for progress in preclinical and clinical applications such as the development of external bioartificial liver (BAL) devices and liver organoid transplantation [5,12,13,14,15]. However, the restricted morphological and functional maturity of iHeps and their lack of long-term functional stability are issues that continue to limit their applicability to regenerative medicine.

During this study, we self-assembled hiPSC-derived liver progenitors and refined the final step of our differentiation protocol [16] to generate hepatocyte organoids (iHep-Orgs). Alongside hepatocyte growth factor (HGF), the iHep-Orgs were exposed to vitamin K and were supplied daily with changing concentrations of dexamethasone (Dex); moreover, oncostatin M (OSM) concentrations were gradually tapered until complete removal. The disappearance of AFP expression and its secretion as well as the ability to fulfil diverse hepatic functions showed that the iHep-Orgs thus generated reached an improved stage of maturation when compared to previously reported data, coming closer to reproducing the functions of primary adult hepatocytes.

## 2. Materials and Methods

### 2.1. hiPSC-Derived Hepatocyte Organoids

Human iPSCs were differentiated into hepatoblasts (iHBs) according to our previously published protocol with some minor modifications [16,17]. Briefly, hiPSCs were seeded on gelatin-coated plates and iHB differentiation was induced using growth factors and cytokines, as reported in the Supplementary Materials and Table S1.

On day 11, the iHBs were harvested (StemPro Accutase Cell Dissociation Reagent; Gibco, Waltham, MA, USA) and processed to induce their self-assembly, differentiation, and maturation into iHep-Orgs. Briefly, non-adherent agarose µ-cylinders (µwells) were created as previously reported [18] using silicone molds. Progenitors were seeded at a density of 3500 iHBs per organoid and incubated at 37 °C under 5% CO_2_. From this point onwards, 20 ng/mL of hepatocyte growth factor (HGF), 0.1 nM of dexamethasone (Dex), and 20 ng/mL of oncostatin M (OSM) were used to induce hepatocyte differentiation. Hepatocyte basal medium (HBM^TM^) complementing the HCM^TM^ bullet kit (Lonza, Walkersville, MD, USA) was used subsequently while omitting hydrocortisone and human epidermal growth factor, and refreshed every other day. On day 18 of the differentiation protocol 10 ng/mL vitamin K1 (Roche, Basel, Switzerland) was introduced and maintained until the end of the culture, alongside 20 ng/mL of HGF, 0.1 nM of Dex, and 20 ng/mL of OSM. From day 20, Dex was administered at an everyday rate which ensured that its supply was reduced to 0.05 nM and then reversed back to 0.1 nM every 24 h until the end of the culture. From day 22, 0.5 nM of Compound E (Santa Cruz Biotechnology, Santa Cruz, CA, USA) and 5 nM of SB431542 (Tocris Biosciences, Bristol, UK) were added to the medium while the OSM concentration was decreased by half every other day (10 ng/mL; 5 ng/mL; 2.5 ng/mL) until its complete removal. The 3D culture of iHep-Orgs was extended until day 38.

### 2.2. In Vitro Assessment of Hepatocyte Functions

Albumin and αFetoprotein secretion. Culture media were collected 24 h after refreshing the medium. AFP levels were measured using the AFP Human ELISA Kit (Fisher Scientific, Waltham, MA, USA) according to the manufacturers’ instructions. Albumin secretion was quantified using the Human Albumin ELISA Quantification Set (Bethyl Laboratories, Montgomery, TX, USA), as instructed.

Bile acid production and transport (excretion). The production of bile acids (BAs) by iHep-Orgs were investigated by liquid chromatography-tandem mass spectrometry (LC-MS/MS) on cell lysates and culture supernatants as previously described [19]. Moreover, the total BA content was quantified in both iHep-Orgs and PHH-Orgs using the Total Bile Acid (TBA) (Human) ELISA Kit (BioVision, Milpitas, CA, USA) in accordance to the manufacturer’s instructions. Moreover, BA transport in iHep-Orgs was investigated by DCFA (5(6)-Carboxy-2′,7′-dichlorofluorescein) (Abcam, Cambridge, UK) metabolism. The samples were incubated with 5-mM probe solutions for 30 min; after washing, images were captured under the microscope (EVOS™ FL Auto Imaging System) in the GFP channel. The iHep-Orgs were then fixed and labeled for the Bile Salt Export Pump (BSEP) apical transporter.

Morphology of bile canaliculi. The image datasets resulting from DCFA assays and immunofluorescence labeling were processed using FIJI software to obtain 3D reconstructions of the bile canaliculi to enable their visualization and measurement of their length. Details are reported in the Supplementary Materials. Three samples were analyzed during six independent experiments.

### 2.3. Hepatocyte Biotransformation Abilities

Phase I metabolism—Oxidative and reductive reactions. To measure cytochrome P450 activity, luminescence-based assays (Promega, Madison, WI, USA) for cytochromes (CYP) 1A1, 1A2, 2B6, 3A7, and 3A4 were used as instructed. Moreover, the activities of CYP1A2 and CYP3A4 were investigated in more detail. Information on the protocols used is given in the Supplementary Materials.

Phase II metabolism—conjugation reaction. Uridine diphospho-glucuronosyl transferase 1A1 (UGT1A1) activity was assessed by quantifying the glucuronidation of 4-Methylumbelliferone (4-MU) (Sigma–Aldrich, St. Louise, MO, USA). iHep-Orgs and PHH-Orgs were treated with 100 µM 4-MU for 24 h then the supernatants were collected and the metabolite was quantified using a fluorescence microplate reader at 450 nm (Spectafluor Plus, TECAN, Männedorf, Switzerland).

Alcohol detoxification. To evaluate alcohol metabolism in iHep-Orgs and PHH-Orgs, the Alcohol Dehydrogenase (ADH) Activity Colorimetric Assay Kit (CliniSciences, Nanterre, France) was used in accordance with the manufacturer’s instructions. iHep-Orgs were tested with and without 10 mM EtOH.

Glucose metabolism. iHep-Orgs and PHH-Orgs were incubated for 4 h with 25 mM D-Glucose and 100 nM insulin to mimic hyperglycemia. Glycogen synthesis and storage were then determined using the Periodic Acid–Schiff (PAS) Staining Kit (Sigma–Aldrich) according to the manufacturer’s instructions. The following day, about 500 organoids were incubated in a glucose-free medium supplemented with 10 nmol/L glucagon (Sigma–Aldrich) in order to mimic short fasting conditions and assess glycogenolysis. Furthermore, to evaluate the capacity for gluconeogenesis, a long fasting state was mimicked by culturing samples without glucose but supplemented with 2 mM of pyruvate for 15 h. The media were then collected, and glucose levels determined using the High Sensitivity Glucose Assay Kit (Sigma).

Lipid metabolism. iHep-Orgs were incubated for 24 h with 0.75 mM of cholesterol lipid concentrate (Gibco), then fixed and cryopreserved. The formation of lipid droplets was then assessed on cryosections (7 µm) using the Lipid (Oil Red O) Staining Kit (Sigma) from both treated and non-treated iHep-Orgs.

Urea production and lactate detoxification under moderate pathological conditions. iHep-Orgs and PHH-Orgs were treated for 4 h with ammonia (1.5 mM) and lactate (2 mM). Urea production and lactate detoxification were assessed using the QuantiChrome Urea Assay Kit (BioAssay Systems, Hayward, CA, USA) and Lactate Assay Kit (Sigma), respectively, according to the manufacturer’s instructions.

## 3. Statistics

The results were expressed as mean +/− standard deviation (SD). Statistical analysis was performed by one-way ANOVA with the Tukey–Kramer test for multiple comparisons. Values were considered to be significant for *p* values of <0.001 (***), <0.01 (**) and <0.05 (*).

## 4. Results

### 4.1. Generation and Characterization of iHep-Orgs

**Figure 1:**
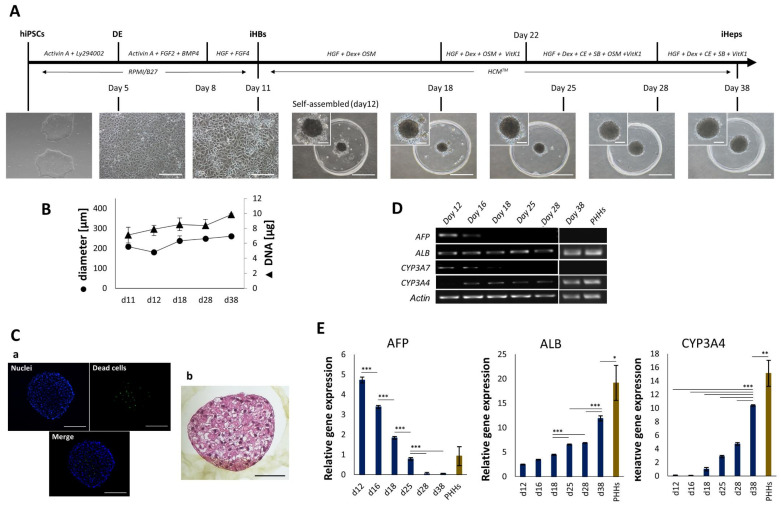
Generation of iHep-Orgs from hiPSCs. (**A**) Schematic representation of the differentiation protocol and brightfield images of undifferentiated hiPSCs, differentiated endoderm (day 5), hepatoblasts (day 11), and self-organized iHep-Orgs after 24 h of assembling (day 12) and over time (days 18, 25, 28, 38). Scale bars for brightfield images = 100 µm. Scale bars for iHep-Orgs = 500 µm. Scale bars for magnifications = 100 µm. (**B**) Evolution of diameter and DNA content of iHep-Orgs over time (measures carried out on 35 and 200 organoids, respectively). Graph represents mean values ± SD (*n* = 16). (**Ca**) Viability assay of iHep-Orgs at day 38 of differentiation. Alive cells’ nuclei are stained in blue; dead cells’ nuclei are stained in green. Scale bars = 100 µm. Images are the Z-projections obtained by combining multiple images taken at different focal distances (z-stacking); (**Cb**) H&E staining on section of a representative iHep-Org. Scale bar = 100 µm. (**D**) RT-PCR analysis of AFP, ALB, CYP3A7, and CYP3A4 gene expression in iHep-Orgs over time and PHH-Orgs at day 8 of culture. (**E**) AFP, ALB, and CYP3A4 gene expression in iHep-Orgs over time (blue bars). PHHs have been used as control (brown bars). Quantification is relative to the expression level in fetal human hepatocytes (GS 20 weeks). Histograms represents mean ± SD (*n* = 16). *** indicates *p* < 0.001; ** indicates *p* < 0.01; * indicates *p* < 0.05.

Our previously published differentiation protocol [16] enabled us to ensure the uniform differentiation of hiPSCs into iHBs (Figure 1A and Supplementary Figure S1A). A homogeneous sheet of endoderm was obtained after five days of treatment with Activin A and LY294002, and the input of FGF2, BMP4, HGF, and FGF4 (Supplementary Table S1) induced the differentiation into hepatic progenitors by day 11. About 98% of endoderm cells were positive for CXCR4 expression at day 5, and 99.4% of the iHB population were positive for expression of the epithelial cell adhesion molecule (EPCAM) by day 11 of culture (data not shown). Gene expression analysis confirmed the disappearance of pluripotent markers alongside the appearance of hepatoblastic markers (Supplementary Figure S1B). At day 11, iHBs displayed a well-defined morphology and markers such as the hepatocyte nuclear transcription factor HNF4α and α-fetoprotein (AFP) appeared to be homogeneously expressed at day 11 (Supplementary Figure S1C). EPCAM and cytokeratin 19 (CK-19) intermediate filament proteins, responsible for the structural integrity of epithelial-like cells, were also expressed in all the cells, as revealed by immunofluorescence analysis (Supplementary Figure S1C). To generate hepatocytes with mature features, iHBs were self-assembled in inert agarose µwells. The cells quickly rearranged themselves into small clusters that fused into a smooth and well-defined iHep-Org within 24 h (day 12) (Figure 1A and Supplementary Figure S1A), whereas PHHs seeded in the same conditions and used as control took 48 h before generating organoids (Supplementary Figure S2). iHep-Orgs retained their shape and compactness over time; minor changes in diameter were observed but no significant changes to DNA content were recorded (Figure 1B) as also confirmed by low Ki67 labeling (data not shown). In particular, after 38 days of culture and despite an average diameter of 250 µm being reached, the organoids were still viable and an absence of necrotic cores was confirmed by H&E staining (Figure 1Ca,Cb). Their gene expression profile analyzed using RT-PCR (Supplementary Figure S3) revealed the expression of hepatic markers as soon as 24 h of 3D culture (day 12); these included mRNAs for α1-antitrypsin (A1AT), asialoglycoprotein receptor (ASGR), HNF4α, low-density lipoprotein receptor (LDL-R), and apolipoprotein AII (APO-A II). mRNAs for HNF1α, bilirubin UDP- glucuronosyltransferase (Bil-UGT), and CYP2B6 were also expressed from days 15, 18, 22, and 28, respectively, thus confirming iHep-Org maturation over time (Supplementary Figure S3). Most remarkably, AFP mRNA expression in iHep-Orgs vanished completely within one week of culture (day 18), while ALB mRNA was already expressed within 24 h. Likewise, a decrease in CYP3A7 mRNA levels and the growing expression of CYP3A4 were recorded (Figure 1D). Quantitative PCR (qPCR) confirmed these findings (Figure 1E). Moreover, the expression profiles showed a loss of AFP mRNA within two weeks of 3D culture, whereas the expression of albumin and CYP3A4 increased over time, with the values recorded at day 38 reaching +65% and +68% of those recorded for PHHs.

### 4.2. Assessment of Hepatic Functions

**Figure 2:**
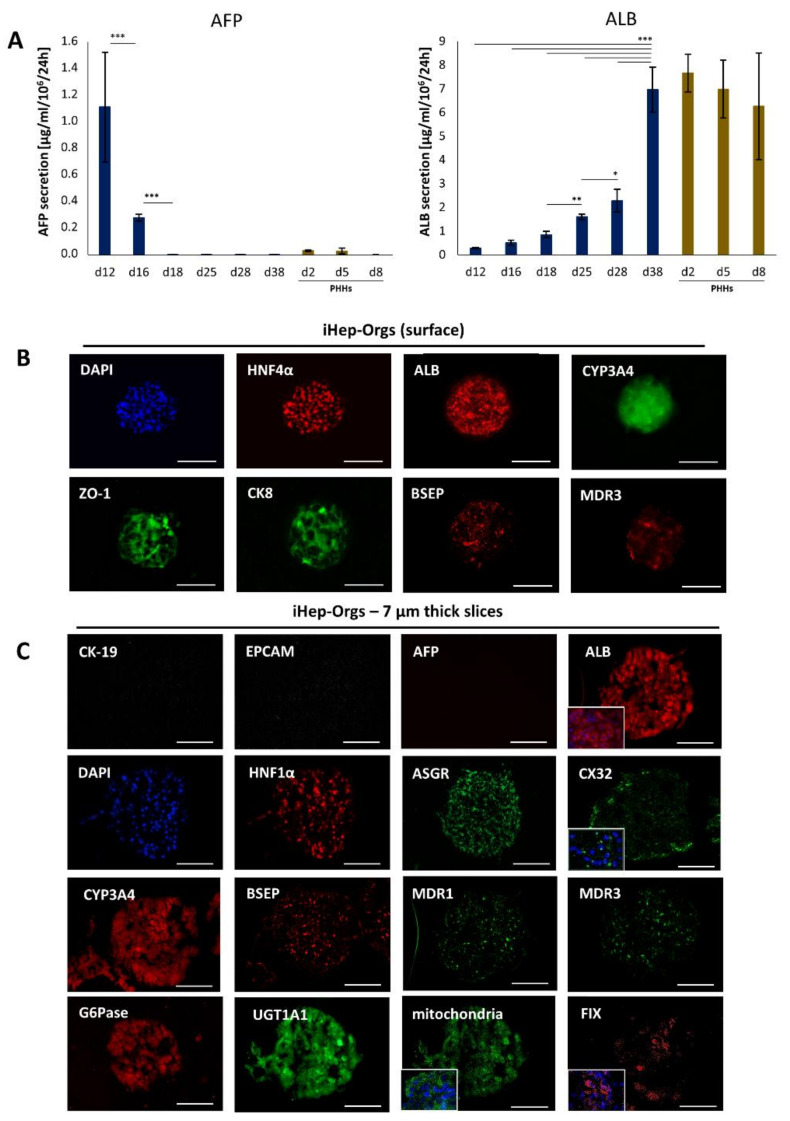
Immunofluorescence staining of iHep-Orgs at the final stage of the differentiation. (**A**) ELISA quantification of AFP and ALB secretion of iHep-Orgs (blue bars) and PHH-Orgs (brown bars). Histograms represent mean ± SD (*n* = 16). *** indicates *p* < 0.001; ** indicates *p* < 0.01; * indicates *p* < 0.05 (**B**) Whole iHep-Orgs immunostained for HNF4α, ALB, CYP3A4, ZO-1, CK-8, BSEP, and MDR3. Nuclei are stained with DAPI. Scale bars = 200 µm. (**C**) Sections (7 µm thick) of iHep-Orgs immunostained for CK-19, EPCAM, AFP, ALB, HNF1α, ASGR, CX32, BSEP, MDR1, MDR3, CYP3A4, UGT1A1, G6Pase, mitochondria, coagulation factor IX (FIX). Nuclei are stained with DAPI. Scale bars = 100 µm.

At the protein level, AFP detection was lost by day 18, while increasing concentrations of ALB were recorded over time (Figure 2A and Supplementary Figure S1D). At day 38, 7 µg/10^6^/24 h of secreted ALB were detected, average value comparable to that recorded for PHH-Orgs (Figure 2A left panel). Immunofluorescence analysis of whole iHep-Orgs (Figure 2B) and iHep-Org cryosections (Figure 2C) showed that the cells were homogeneously differentiated into hepatocytes. Indeed, biliary markers as CK-19 and EPCAM were no more expressed while hepatocyte markers were expressed uniformly on both the surface and inside the iHep-Orgs. Moreover, the distribution of BSEP and multidrug resistance proteins (MDR1 and MDR3), concomitant with the gap-junction protein CX32 expression, highlighted the acquisition of the typical hepatic complex basolateral-apical polarization and the formation of a bile canaliculi. In addition, the labeling of UGT1A1, coagulation factor IX (FIX), glucose 6-phosphatase (G6Pase), and mitochondria confirmed that iHep-Orgs had acquired an improved degree of maturation.

### 4.3. Bile Acid Secretion and Bile Canalicular Network in iHep-Orgs

**Figure 3:**
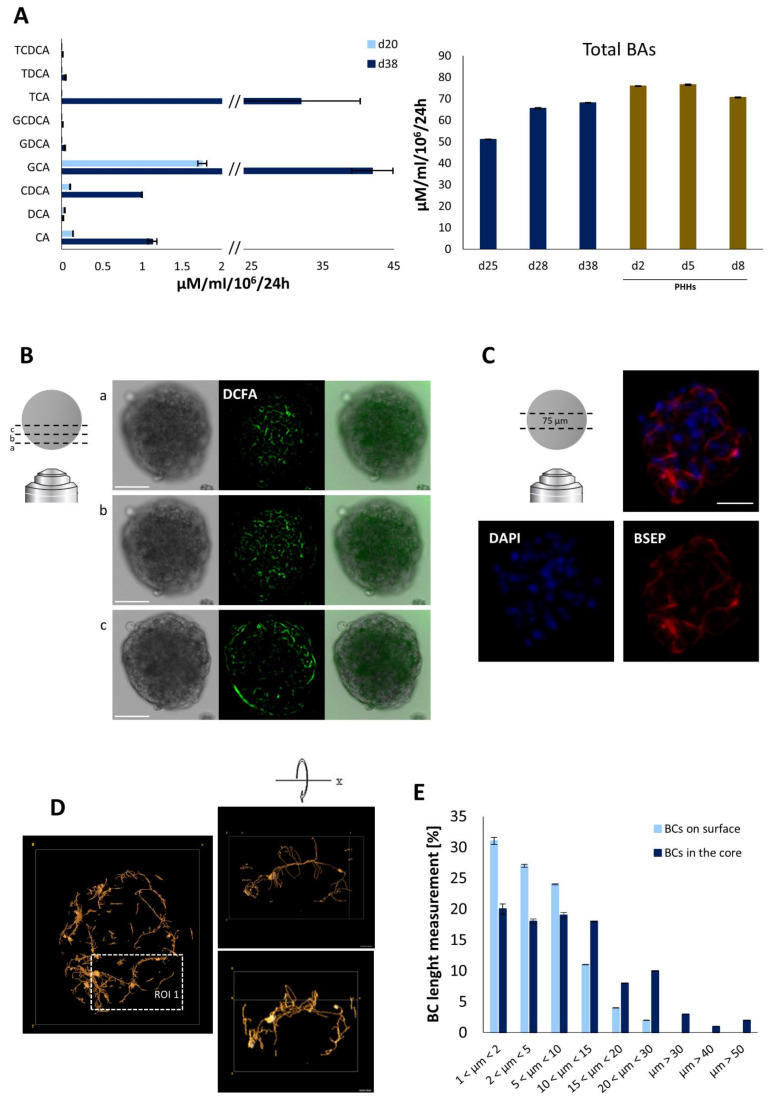
Bile acid secretion and bile canaliculi network analysis of iHep-Orgs. (**A**) Left panel: bile acid production and secretion by iHep-Orgs in culture supernatants at day 20 (light blue bars) and 38 (blue bars) of culture. Histograms represent mean ± SD (*n* = 3). CA = cholic acid; DCA = deoxycholic acid; CDCA = chenodeoxycholic acid; GCA = glycocholic acid; GDCA = glycodeoxycholic acid; GCDCA = glycochenodeoxycholic acid; TCA = Taurocholic acid; TDCA = taurodeoxycholic Acid; TCDCA = taurochenodeoxycholic acid. Right panel: total bile acid production and secretion by iHep-Orgs (blue bars) and PHH-Orgs (brown bars) over time. Histograms represent mean ± SD (*n* = 3). (**B**) Live imaging of the bile canaliculi network formed by excretion of the fluorescent probe DCFA in iHep-Org at day 38. Scale bar = 100 µm. (**C**) Immunofluorescence staining for the apical membrane transporter BSEP in iHep-Org sections (75 µm) at day 38. Scale bar = 75 µm. (**D**) Left panel: 3D reconstruction of the BC network from the apical membrane transporter BSEP staining in iHep-Org sections (75 µm) at day 38. Right panel: 3D reconstruction of a segment (ROI 1) of the BC network (rotation in *x* axis). (**E**) Quantification and length measurements of bile canaliculi recorded on the surface (light blue bars) and in the core (blue bars) of the iHep-Orgs after DCFA treatment and BSEP staining. Three iHep-Orgs from each of 6 independent experiments were analyzed.

BA production and secretion were detected in iHep-Orgs already by day 20 (Supplementary Figure S4A) in both cell lysates and culture supernatants. Cholic acid (CA) and chenodeoxycholic acid (CDCA) were indeed secreted at three weeks of culture, alongside glycocholic acid (GCA). By day 38, a significant increase was observed in the secretion of CA and CDCA, the major primary bile acids synthesized in the human liver, and their conjugated forms with taurine (TCA and TCDCA) or glycine (GCA and GCDCA) (Figure 3A, left panel). These findings thus attested to the correct expression and activity of both microsomal enzyme cholesterol 7α-hydroxylase (CYP7A1) and bile acid coenzyme A: amino acid N-acyltransferase (BAAT). Moreover, in terms of total BA secretion, no significant differences were recorded between the metabolic capacities of iHep-Orgs and PHH-orgs as of day 28 (Figure 3A, right panel). To characterize iHep-Org in greater detail, basolateral/apical transporter activity and the formation of bile canaliculi were also investigated. First, an indocyanine green (ICG) uptake/release study revealed that iHep-Orgs were able to uptake the probe within 15 min, thus evidencing the OATP1B3 (organic anion-transporting polypeptide 1B3) and NTCP (Na^+^-taurocholate co-transporting polypeptide) expression (Supplementary Figure S4B). Likewise, the complete excretion of dye in less than 3 h indicated activity of the apical transporter MRP2 (multidrug resistance-associated protein 2) at the apical pole of the hepatocytes. Second, using the fluorescent probe DCFA, which is also a tracer of MRP2 transport, we observed the development of a network of elongated bile canaliculi (BCs) at the surface of the iHep-Orgs (Figure 3B). In view of these results, we wondered if the BC network might extend to the core of the organoids. We therefore labeled 75-µm-thick slices of the DCFA-treated iHep-Orgs for BSEP expression and analyzed the datasets obtained from imaging. The Z-stack reconstruction did indeed confirm our hypothesis. As shown in Figure 3C, BCs looked as well-defined and finely organized structures that appeared to involve a fair number of cells. Images and representative regions of these slices were also further processed to obtain 3D reconstructions of the network thus highlighted. This in-depth investigation clearly showed that a three-dimensional and complex system of branching canaliculi extended within the organoids (as shown in Figure 3D for ROI 1). Measurements of the BC revealed that this network was mostly made up of 2 µm-, 5 µm-, and 10 µm-long canaliculi (82%) on the iHep-Org surface and only 6% of the structures far exceeded these lengths. By contrast, in the organoid cores, the percentage of small branches shifted significantly (57%) towards longer structures. In particular, 6% of the BC detected were longer than 40 µm (Figure 3E).

### 4.4. Biotransformation Capabilities of iHep-Orgs

**Figure 4:**
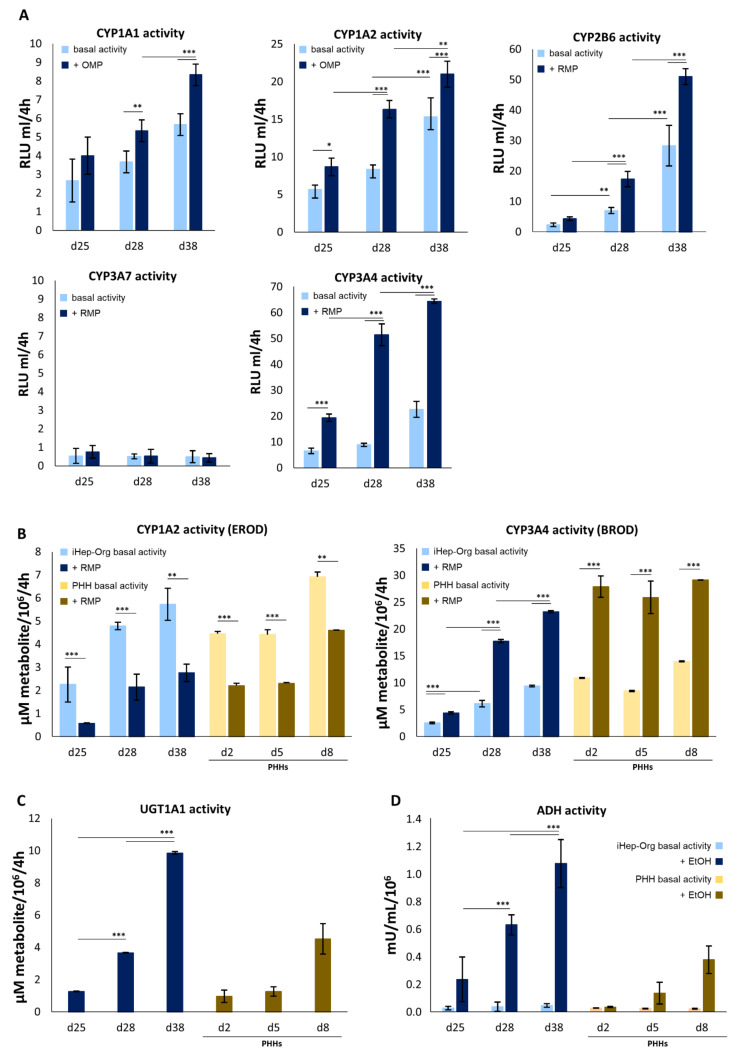
Activity of phase I and II metabolisms of iHep-Orgs. (**A**) CYP1A1, CYP1A2, CYP2B6, CYP3A7 and CYP3A4 activities measured in iHep-Orgs without (light bars) and after induction (blue bars). Histograms represent mean ± SD (*n* = 3). (**B**) CYP1A2 (EROD) and CYP3A4 (BROD) specific activities of iHep-Orgs (blue shade bars) and PHH-Orgs (brown shade bars). Histograms represent mean ± SD (*n* = 8). (**C**) UGT1A1 activity of iHep-Orgs (blue bars) and PHH-Orgs (brown bars). Graph represents mean ± SD (*n* = 6). (**D**) ADH activity of iHep-Orgs (blue shade bars) and PHH-Orgs (brown shade bars). *** indicates *p* < 0.001; ** indicates *p* < 0.01; * indicates *p* < 0.05.

In order to achieve a broad characterization of the hepatocytic function in iHep-Orgs, we turned to investigating phase I and II detoxification enzyme activities and their responsiveness to hormones, glucose deprivation and alcohol. The enzymatic activities of CYP1A1, 1A2, 2B6, 3A7, and 3A4 were examined to evaluate xenobiotic phase I metabolism. As expected, iHep-Orgs already displayed detoxification capabilities by day 25, and their performance improved over time. Only CYP2B6 activity appeared later on, thus confirming the data obtained from gene expression analysis. Moreover, all the isoforms investigated responded to the induction treatment (Figure 4A). Significantly, the activity of CYP3A7, the fetal isoform of CYP3A4, was the only enzyme whose recorded activity values were close to zero, according to the RT-PCR results. This again indicates that the iHep-Orgs had reached a significant level of maturation (Figure 4A). To obtain more detail and compare our organoids with PHH-Orgs, CYP1A2, and CYP3A4 activities were also specifically examined using EROD and BROD specific tests. Both isoforms in iHep-Orgs were able to metabolize the drug supplied and responded to rifampicin treatment by a 50% reduction and 60% increase in CYP1A2 and CYP3A4 detoxification capabilities, respectively (Figure 4B). Further confirmation of the high level of functional maturation achieved by our organoids, their metabolic capacities closely mirror those of PHH-Orgs.

Glucuronidation through UGT1A1 (phase II metabolism) and the detoxification of ethanol by alcohol dehydrogenase (ADH) are specific features of adult hepatocytes. Hepatocyte organoids treated with 4-MU and EtOH displayed both UGT1A1 and ADH activity as early as day 25 of culture, and a significant increase in detoxification capabilities over time. At day 38 of differentiation, a 3-fold higher concentration of conjugated metabolite was detected (Figure 4C) and importantly, the iHep-Orgs already displayed increased ADH activity by day 25 of culture (Figure 4D), thus proving them to be capable of metabolizing ethanol in an inducible manner. Unexpectedly, as early as day 25, iHep-Orgs proved to be functionally comparable or superior to PHH-Orgs for both enzymatic activities.

**Figure 5:**
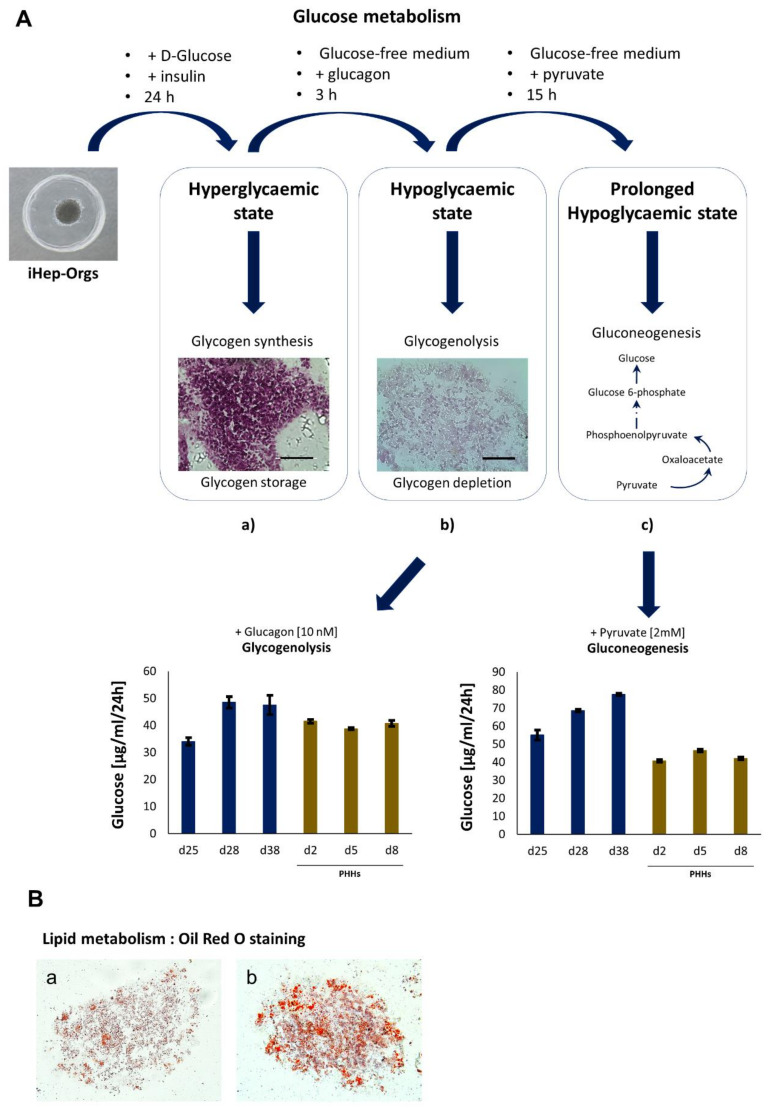
Metabolism in iHep-Orgs. (**A**) Scheme depicting the glucose metabolism analysis in iHep-Orgs. (**a**,**b**) Periodic acid–Schiff staining of glycogen in iHep-Orgs under hyperglycemic and hypoglycemic conditions, respectively. Scale bar = 50 µm. (**c**) Representation of the gluconeogenesis process from pyruvate in hepatocytes. Graph of glucose quantification after glycogenolysis (bottom left) and gluconeogenesis (bottom right) in iHep-Orgs (blue bars) and PHH-Orgs (brown). (**B**) Lipid metabolism in iHep-Orgs. Oil Red O’ staining of lipid droplets in (**a**) iHep-Orgs cultured in normal conditions and (**b**) iHep-Orgs cultured in presence of high lipid concentration.

Hepatocytes are crucial for the maintenance of normal glucose homeostasis, so we investigated the glycogenolysis and gluconeogenesis capabilities of the iHep-Orgs as they mimicked in vitro both a short-lived and lengthy fasting state (Figure 5A). Under hyperglycemic conditions and insulin stimulation, the iHep-Orgs were able to respond through glucose anabolism, as assessed by the glycogen storage observed (Figure 5Aa). Moreover, in the absence of glucose (hypoglycemic state) and in the presence of glucagon, the organoids were also able to catabolize the glycogen accumulated over the previous 24 h and then release it as glucose into the medium (Figure 5Ab). Likewise, iHep-Orgs responded to a prolonged hypoglycemic state. Using pyruvate as the only source available, they activated gluconeogenesis and released glucose into the medium (Figure 5Ac). As for UGT1A1 and ADH activities, iHep-Orgs also proved to be functionally comparable or superior to PHH-Orgs in both glycogenolysis and gluconeogenesis performances. Lipid metabolism also plays a central role in liver function, so we assessed iHep-Org ability in storing fatty acids as lipid droplets. Again, the organoids were shown to be capable of metabolizing fatty acids and forming lipid droplets, whose quantity and size were visibly increased when the organoids were cultured in the presence of high lipid concentrations (Figure 5B).

**Figure 6:**
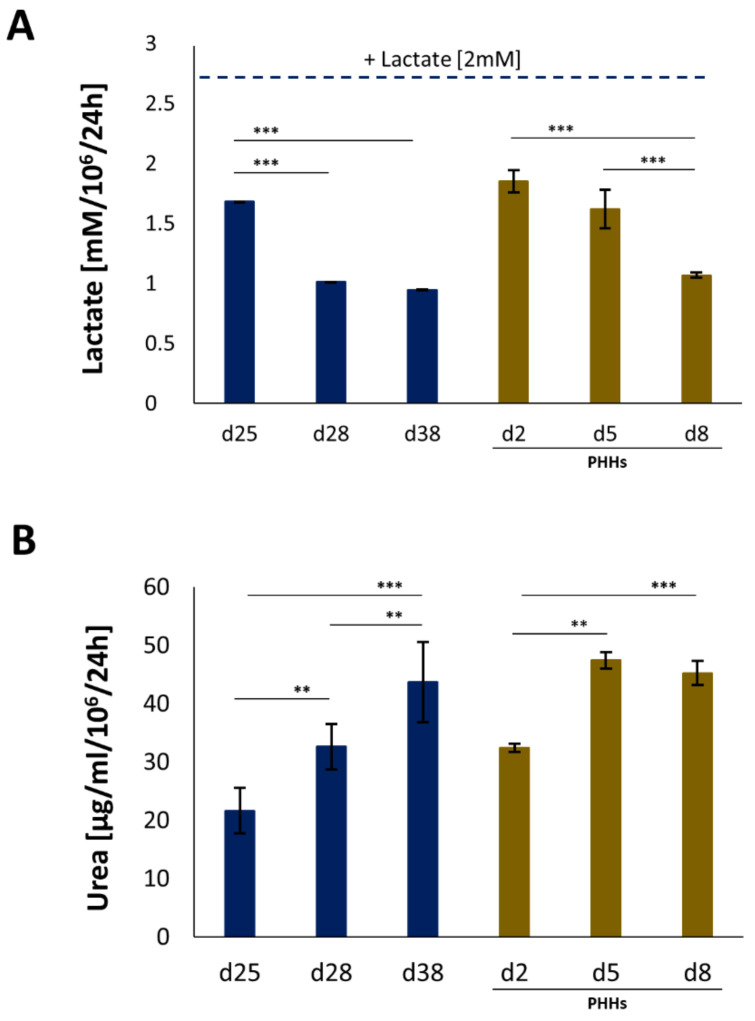
Detoxification abilities of iHep-Orgs in moderate pathological conditions. (**A**) Lactate detoxification and (**B**) urea synthesis of iHep-Orgs (blue bars) and PHH-Orgs (brown bars). Graphs represent mean ± SD (*n* = 8). *** indicate *p* < 0.001; ** indicate *p* < 0.01.

The ability to detoxify lactate and ammonia is one of the major features mandatory to enable the use of external BAL devices to treat the plasma of patients suffering from acute or acute-on-chronic liver failure. We therefore mimicked a pathological environment to challenge their detoxification capabilities. More specifically, 2 mM of lactate and 1.5 mM of ammonia were added to the medium. By day 25, the iHep-Orgs were able to detoxify both compounds; lactate levels were restored to normal after 24 h, while ammonia was converted into urea (Figure 6). Data recorded were, once again, comparable or superior to PHH-Orgs. Remarkably, despite the iHep-Orgs having been treated for two consecutive weeks, not only did they sustain their metabolic activity, but the detoxification rate had increased by 40% and 50% for lactate metabolism and urea synthesis, respectively by the end of culture (day 38) (Figure 6).

## 5. Discussion

During the past decade, considerable progress has been made in optimizing hiPSC differentiation techniques, and the efficiency of these protocols now reaches 90% [20], enabling the production of iHeps that can be used under both 2D and 3D configurations to study liver development, diseases and the efficacy of medicinal products. However, so that iHeps can be used as a cell source to better predict drug-induced hepatotoxicity/efficacy and for cell-based applications in the clinical setting, promoting functional maturation remains a major challenge. Indeed, iHeps currently produced retain fetal characteristics including limited detoxification efficacy.

When it comes to differentiating and characterizing iHeps in vitro, there are at least two important factors that must be considered. First, it is well known that the liver microenvironment changes significantly after birth, inducing the maturation of fetal/neonatal cells into fully functional adult hepatocytes. The complexity of these signals has not yet been deciphered so it cannot be completely reproduced in vitro. Second, choosing the most appropriate tests to investigate the functional maturation of cells is not simple, nor is the choice of pertinent controls. As well as morphological changes, mature hepatocytes in vivo acquire responsiveness to hormones, an ability to maintain glucose and ammonia homeostasis and detoxification capabilities. The in vitro culture of PHHs has drawbacks such as a loss of function: cryopreserved PHHs do not display the same metabolic capabilities as freshly isolated ones, and the latter suffer from limited availability and incongruous functions due to significant differences in donor background. Therefore, although transcriptomic analysis has enabled the comparison of iHep gene expression patterns and those of PHHs [21], and indeed revealed 80% similarity between them, differences in cell sources, data, in vitro culture methods and analytical procedures have produced different and controversial results [22,23,24,25]. Based on these widely acknowledged problems, the present study was designed to improve the maturation of iHep-Orgs so that they could become a reliable tool to progress in pharmacological/toxicology studies as well as preclinical and clinical trials of external bioartificial liver (BAL) devices and pluripotent stem cell-based clinical applications. To achieve this, we broadened the scope of functional characterizations including tests that are not used frequently in in vitro models, and directly compared iHep-Org functional features to PHH organoids.

The refinements made to our protocol were based on (i) the slow withdrawal of the OSM, a member of the IL-6 family secreted by hematopoietic cells proliferating in the fetal liver. Indeed, OSM strongly enhances differentiation of fetal hepatocytes during liver organogenesis [26] but, at the end of the second trimester of the gestation, its concentration declines with the migration of hematopoietic cells from the fetal liver to the bone marrow alongside allowing the hepatocyte maturation. To mimic this step of the liver organogenesis, we modified our protocol to progressively suppress OSM in the culture medium from day 21 when iHeps have reached the fetal hepatocyte stage. (ii) Second, we relied on the fact that it was shown that glucocorticoids supplement, in particular through dexamethasone, highly affects the maturation of fetal and neonatal hepatocytes towards adult hepatocytes contributing to the switch of connexin 26 in connexin 32 and the upregulation of the transcript levels [27,28]. These gap junction proteins (GJPs) are indeed known to be indispensable for cell-cell communications so that glycogenolysis, albumin secretion, ammonia detoxification, CYP-mediated xenobiotic biotransformation, and bile secretion are correctly carried out [29]. In addition, it has been demonstrated that the regulation of the GJPs partially revolves around the Dex ability to stimulate vitamin K-dependent carboxylation process in fetal hepatocytes in culture. (iii) We thus proceeded with the regular administration of vitamin K1, the major dietary source and primary circulating form of vitamin K, during our maturation protocol, alongside the fine regulation of the glucocorticoid supplement. Vitamin K (Vit K) is a group of fat-soluble vitamins which act as indispensable cofactors in the post-translational γ-carboxylation of glutamic acid residues of coagulation-associated proteins such as factors II (prothrombin), VII, IX, and X [30,31]. It proved, in animal models, to be accountable for the regulation of the signaling pathways of inflammation (NF-κB) processes, glucose metabolism (SIRT1/AMPK/PI3K/PTEN/GLUT2/GK/G6Pase), and lipid oxidation (PPARα/CPT1A) [32,33]. Therefore, the correct supplement of Vit K can be crucial in determining the acquisition of a high level of maturation by hepatocytes in culture. Indeed, we developed an in vitro model of hemophilia B hepatocytes using iPSCs generated from fibroblasts of a severe HB patient. Through the 3D culture and by optimizing the maturation step of the differentiation protocol, we produced iHeps secreting the active form of the coagulation factor IX, specific ability of adult hepatocytes [34].

The supply of vitamin K1, the modulation of dexamethasone concentrations and the removal of OSM improved the differentiation of iHeps; indeed, combined with 3D culture, iHep-Orgs proved to be very similar in function to PHH-Orgs. A direct comparison to literature reported data from PHHs is difficult, due to their heterogenicity especially in terms of albumin secretion or CYP450 activities (Table S4); nevertheless, we found our findings worthy of attention for the complete disappearance of AFP expression and secretion by iHep-Orgs. Indeed, this is a hallmark widely used to discriminate fetal and adult hepatocytes, so the loss of AFP expression testifies them achieving a functional maturity that to our knowledge, has not previously been reported [35,36,37,38,39] (Table S4). Two independent clones of hiPSCs were tested and the results obtained, shown in Supplementary Figure S1, confirm not only the same ability to differentiate into hepatoblasts but also and especially the same trend in terms of AFP disappearance to the benefit of ALB secretion. The generated iHep-Orgs, when exposed to the maturation conditions presented in this work, are indeed capable of reaching a significant level of maturation confirming the robustness of the new protocol.

Another important marker of the improved maturation of iHeps is the extinguished expression of the fetal enzyme CYP3A7 mRNA with the concurrent appearance of CYP3A4 mRNA expression (Figure 1D). Further confirmation that iHep-Orgs were nearly mimicking primary adult hepatocytes in terms of their morphology and functions was provided by the expression of different proteins and the investigation of metabolic activity. Immunofluorescence staining revealed a homogeneous distribution of hepatocyte markers on both the surface of iHep-Orgs and in the core of the organoids. Alongside the typical markers of hepatic differentiation (i.e., albumin, HNF4α and 1α, CYP3A4, and CK-8) the iHep-Orgs also displayed UGT1A1, connexin 32 (CX32), and G6Pase (whose expression is essential for hepatic functions such as glycogenolysis), coagulation factor IX, and a significant number of mitochondria, as might be expected in cells with high metabolic activity such as hepatocytes.

Bile acid production, in concentrations similar to those of PHHs, and their secretion proved that the hepatocytes within iHep-Orgs have acquired a high degree of maturation and the expected complex polarity. The distribution of the apical markers BSEP and MDR1, concomitant with the expression of ZO1, both on the surface of the organoids and into the structures (Figure 2B,C and Figure 3C) suggest the presence of a bile canalicular network developed enough to connect the core of the structures with their surface. This would allow drainage of the produced bile outward (into the culture medium), on which the absence of a necrotic core and the viability of iHep-Orgs would consequently depend (Figure 1C). Although some published protocols have confirmed the expression of apical markers in human iHeps, to our knowledge, no study as yet had assessed development of the BC network into the core of hepatic organoids. Finally, the ability of iHep-Orgs to fulfil some of the most important adult liver functions, further testify to the robust and sustained maturation of our iHeps cultured as organoids until at least day 38 and even up to lastly tested day 45. For phase I and II metabolisms, alcohol metabolism, a hormone-induced response to hyperglycemic and hypoglycemic conditions, iHep-Orgs showed results comparable to, and in some cases even above, those of PHH-Orgs, as well as a remarkable detoxification ability. Indeed, in a pathological environment, iHep-Orgs were shown to be able to metabolize lactate and ammonia, thus markedly lowering their levels in the culture medium.

Taken together, these results indicate that in view of their potential nearness to reproducing the functions of PHHs, the iHep-Orgs we have generated may prove discriminatory in the development of new liver models for physiology and pathophysiology studies, and the investigation of drug-induced liver injury (DILI). Last but not least, we consider it possible that in the near future it may be possible to use iHep-Orgs for the preclinical testing of bioartificial devices and/or transplantation to treat animals affected by acute or chronic liver failure in potential anticipation of their use in the clinic.

## 6. Conclusions

The correct maturation of iHeps and their phenotypic stability are of considerable importance to their application. Overall, the iHep-Orgs we generated represent a homogenous cell population with close similarity to primary adult hepatocytes and might represent an important tool for the development of new liver models for either liver diseases or pharmaco-toxicology studies, tissue engineering, and regenerative applications.

## Supplementary Materials

The following supporting information can be downloaded at: https://www.mdpi.com/article/10.3390/cells11030537/s1. Figure S1: Assessment of hiPSC differentiation into iHBs; Figure S2: PHH self-assembling into organoids in agarose µwells; Figure S3: Gene expression of hepatocyte markers; Figure S4: Assessment of iHep-Org functions associated with polarization; Table S1: Protocol of hiPSC differentiation into hepatoblasts; Table S2: List of primer sequences used for gene expression analysis; Table S3: List of primary antibodies used for immunofluorescence staining; Table S4: Summary table of the hepatocyte functional features.

## Abbreviations

µwells: micro wells; 4-MU: 7-hydroxy-4-methylcoumarin; A1AT: alpha 1-antitrypsin; ADH: alcohol dehydrogenase; AFP: α-fetoprotein; ALB: albumin; APO-AII: apolipoprotein AII; ASGR: asialoglycoprotein receptor; BAL: bioartificial liver; BAs: bile acids; BCs: bile canaliculi; Bil-UGT: bilirubin UDP-glucuronosyltransferase; BMP4: bone morphogenetic protein 4; BSEP: bile salt export pump; CA: cholic acid; CDCA: chenodeoxycholic acid; CK-8: cytokeratin 8; CK-19: cytokeratin 19; CX32: connexin 32; CXCR4: C-X-C chemokine receptor type 4; CYP1A1: cytochrome P450 1A1; CYP1A2: cytochrome P450 1A2; CYP2B6: cytochrome P450 2B6; CYP3A4: cytochrome P450 3A4; CYP3A7: cytochrome P450 3A7; CYP7A1: cytochrome P450 7A1 or cholesterol 7α-hydroxylase; DCA: deoxycholic acid; DCFA: 5(6)-carboxy-2′,7′-dichlorofluorescein; DE: definitive endoderm; Dex: dexamethasone; EPCAM: epithelial cell adhesion molecule; ETOH: ethanol; FGF2: fibroblast growth factor 2; FGF4: fibroblast growth factor 4; FIX: coagulation factor IX; G6Pase: glucose 6-phosphatase; GCA: glycocholic acid; GCDCA: glycochenodeoxycholic acid; GDCA: glycodeoxycholic acid; GJPs: gap junction proteins; H&E: haematoxylin and eosin; HCM: hepatocyte complete medium; HGF: hepatocyte growth factor; hiPSCs: human induced pluripotent stem cells; HNF1α: hepatic nuclear factor 1 α; HNF4α: hepatic nuclear factor 4 α; ICG: indocyanine green; iHBs: hepatoblasts; iHep-Orgs: hepatocyte organoids; iHeps: human iPSC-derived hepatocytes; LDL-R: low-density lipoprotein receptor; MDR1: multidrug resistance protein 1; MDR3: multidrug resistance protein 3; MRP2: multidrug resistance associated protein 2; NTCP: Na^+^-taurocholate co-transporting polypeptide; OATP1B3: organic anion-transporting polypeptide 1B3; OSM: oncostatin M; PAS: periodic acid–Schiff; PHHs: human primary hepatocytes; qRT-PCR: quantitative real-time polymerase chain reaction; RMP: rifampicin; ROI: region of interest; RT-PCR: real-time polymerase chain reaction; SSEA4: stage-specific embryonic antigen-4; TCA: taurocholic acid; TDCA: taurodeoxycholic acid; TCDCA: taurochenodeoxycholic acid; UGT1A1: uridine diphospho-glucuronosyltransferases 1A1; ZO-1: zona-occludens 1.
