# Supplementary material for: Evidence of Adult Features and Functions of Hepatocytes Differentiated from Human Induced Pluripotent Stem Cells and Self-Organized as Organoids
Source: Cells. 2022 Feb 4;11(3):537. doi: 10.3390/cells11030537 (PMC8834365; doi:10.3390/cells11030537)
Supplement: Supplementary file 1 [file cells-11-00537-s001.zip › cells-1576047-supplementary.pdf]

Evidence of adult features and functions of hepatocytes differentiated from human induced pluripotent stem cells and self-organized as organoids

Antonietta Messina[1,2*], Eléanor Luce[1,2+], Nassima Benzoubir[1,2+], Mattia Pasqua[2,3], Ulysse Pereira[2,3], Lydie Humbert[4], Thibaut Eguether[4], Dominique Rainteau[4], Jean-Charles Duclos-Vallée[1,2], Cécile Legallais[2,3], and Anne Dubart-Kupperschmitt[1,2*]

1- UMR_S 1193, INSERM/Université Paris-Saclay, F-94800 Villejuif, France

2- Centre Hépatobiliaire, Fédération Hospitalo-Universitaire (FHU) Hépatinov, AP-HP, Hôpital Paul Brousse, F-94800 Villejuif, France

3- UMR CNRS 7338 Biomechanics & Bioengineering, Université de Technologie de Compiègne,

Sorbonne Universités, 60203 Compiegne, France

4- Centre de Recherche Saint-Antoine, Sorbonne Université, INSERM, CRSA, AP-HP, Hôpital Saint Antoine, Metomics, 75012 Paris, France

Table of contents

Supplemental Materials and Methods

- Human induced pluripotent stem cells
- Human iPSC differentiation into hepatoblasts
- Organoid's viability assay
- Primary human hepatocytes
- Nucleic acid extraction and gene expression assay
- H&E and immunofluorescence staining
- Indocyanine green (ICG) uptake and release
- Measurement of cytochrome P450 activity
- Morphological analysis of bile canaliculi

Supplemental Tables

- Table ST1. Protocol of hiPSC differentiation into hepatoblasts
- Table ST2. List of primer sequences used for gene expression analysis
- Table ST3. List of primary antibodies used for immunofluorescence staining
- Table ST4. Summary table of the hepatocyte functional features

Supplemental Figures

- Fig. S1. Assessment of hiPSC differentiation into iHBs.
- Fig. S2. PHH self-assembling into organoids in agarose µwells
- Fig. S3. Gene expression of hepatocyte markers.
- Fig. S4. Assessment of iHep-Org functions associated with polarization.

Supplemental Materials and Methods

Human induced pluripotent stem cells

Human iPSC lines were generated and characterized as described in Steichen et al 2014 (1) (Fig. S1). Briefly, human foreskin fibroblast (ATCC CRL2097 – passage 6) were reprogrammed by the daily transfection with mRNAs produced in the laboratory, for 16 days.

Human iPSC differentiation into hepatoblasts

Human iPSC-derived hepatoblasts (iHBs) were obtained as follows. Briefly, hiPSC colonies, cultured on Geltrex™ (Gibco) coated petri dishes, were detached enzymatically using 0.5% Trypsin-EDTA 1X solution (Gibco), seeded at a density of $4.2x10^4$ cells/cm^2 on gelatin-coated plates and incubated at 37°C under 5% CO_2 in StemMACS™ iPS-Brew XF medium with 10 nM Y-27632. Once 70% of confluence was reached, the differentiation protocol was started using RPMI-1640 medium (Gibco) with B-27 serum-free supplement (Life technologies), 1X MEM non-essential amino acid solution (NEAA, Gibco) and 1X penicillin-streptomycin (Gibco). To induce endoderm differentiation, the cells were treated with 5 nM CHIR99021 (Stemcell™ Technologies) for one day and 100 ng/ml Activin A (Miltenyi Biotech) and 10 nM LY294002 (Sigma-Aldrich) for the next four days. From day 5 to day 7, 50 ng/ml Activin A, 10 ng/mL BMP4 (R&D Systems) and 20 ng/mL FGF2 (Miltenyi Biotech) were supplied to specify the hepatic endoderm. From day 8 to day 10, which generated a homogeneous monolayer of differentiated iHBs, 20 ng/ml HGF (Miltenyi Biotech) and 30 ng/ml FGF4 (Miltenyi Biotech) were added to produce a homogeneous monolayer of differentiated iHBs.

Organoid viability assay

iHep-Orgs were recovered on day 38 of culture from the non-adherent agarose µ-cylinders (µwells) using DURAN® disposable micropipettes (Hirschmann Laborgeräte GmbH & Co. KG). They were then washed with 1X DPBS (Gibco) and tested for viability with the Ready Probes® cell viability imaging kit (Molecular Probes®) according to the manufacturer's instructions.

Primary human hepatocytes

Cryopreserved PHHs (Biopredic international) have been thawed according to the provider's instructions, and processed as for self-assembling of iHBs. Spheroids were obtained after 48h, the medium was refreshed every other day and PHH organoids (PHH-Orgs) were maintained in culture for 8 days to be used as control for the functional assessment of iHep-Orgs.

Nucleic acid extraction and gene expression assay

At established time points throughout the culture period, the total RNA/DNA content of iHep-Orgs, fetal human hepatocytes (FHHs) and PHHs was extracted using TRIzol™ Reagent (Sigma-Aldrich Aldrich) and purified with the Direct-zol™ DNA/RNA MiniPrep Kit (Zymo Research) following the manufacturer's instructions. The quantification of RNA and DNA in the samples was performed by UV-visible Nanodrop Lite (ThermoFisher). The DNA contents were used to define the number of cells per sample, so that the

quantification assay results could be shown as value/10^6/24 h if not otherwise specified. Reverse transcriptase-PCR (RT-PCR) was performed using the SuperScript™ First-Strand Synthesis System for RT-PCR (Invitrogen) with oligoDT primer and Platinium Taq DNA Polymerase (Invitrogen), according to the manufacturer's instructions. For quantitative PCR analysis, the mRNA extraction and RTqPCR conditions were set in line with the manufacturer's instructions. cDNAs were obtained using the SuperScript III kit (Invitrogen) with random hexamers. Three replicates per sample were analyzed for differential gene expression using the Mx3000P qPCR thermocycler system (Agilent) with brilliant III ultrafast SYBR Green (Agilent). Relative levels of expression were determined using the $2^{-\Delta\Delta Ct}$ method with GAPDH as the reference gene, and expression levels were described relative to FHHs. A list of primer sequences is shown in Supplementary Table ST2.

H&E and immunofluorescence staining

For histochemical staining, slices of iHep-Orgs (d38) were fixed in 4% PFA for 15 minutes then stained with Hematoxylin and Eosin (Sigma-Aldrich). For immunofluorescence labelling, whole iHep-Orgs and their cryosections (7 µm) were treated according to the same protocol with appropriate adaptations. The slices were permeabilized with 0.1% Triton-X solution supplemented with 1% BSA and 0.2 µg/ml EDTA for 5 minutes on ice before being saturated with 3% BSA solution and treated with primary antibodies overnight. Secondary antibodies were incubated for 1h at RT and the nuclei were stained with DAPI. Whole 3D-iHeps were fixed for 4h in 4% PFA, washed extensively and permeabilized for 45 minutes. A 3-hour saturation step with 5% BSA was carried out before incubation with primary and secondary antibodies for 48 h (4°C) and 3h (RT), respectively; DAPI was used for nuclear staining. Images were captured using the EVOS™ FL Auto Imaging System and a confocal microscope. A list of primary antibodies is shown in Supplementary Table ST3.

Indocyanine green (ICG) uptake and release

To monitor Indocyanine green (ICG) uptake and excretion, iHep-Orgs were treated with 5 µM Cardiogreen (Sigma-Aldrich) solution for 15 minutes at 37°C. The Cardiogreen solution was removed and images were captured under an inverted light microscope (EVOS™ FL Auto Imaging System) immediately and then after 30, 60, and 180 minutes, following rapid washing to remove the excreted ICG.

Measurement of cytochromes P450 activity

Luminescence-based assays (Promega, Madison, WI) for cytochromes P450 (CYP450) 1A1, 1A2, 3A7, 3A4, and 2B6 were used to determine CYP450 activity. Briefly, about 50 iHep-Orgs were incubated at 37°C with luminogenic (luciferin) cytochrome P450 substrate for 4h. The supernatants were collected and luciferase was quantified according to the manufacturer's instructions. Further, in order to assess the inducibility of CYP450 isoforms in iHep-Orgs, 25 µM rifampicin (RMP) (an inducer of CYP3A4, CYP3A7 and CYP2B6) or 100 µM omeprazole (OMP) (an inducer of CYP1A1 and CYP1A2) were added to the culture medium 48h before the test. The supernatants were collected and processed as indicated above. This analysis produced mean luciferase values obtained during three independent experiments.

To enable specific measurement of the activities of CYP1A2 and CYP3A4, iHep-Orgs and PHH-Orgs were treated with 8μM 5-ethoxyresorufin (EROD test) or 8μM 7-benzyloxyresorufin (BROD test), which are respectively the substrates for the two isoforms. To inhibit phase II CYP450 enzyme interference, 3mM salicylamide (Sigma-Aldrich) and 10μM dicoumarol (Sigma-Aldrich) were also added. The supernatants were collected and the metabolite, resorufin, was quantified using a fluorescence microplate reader at 595 nm (Spectafluor Plus, TECAN). 10 μM rifampicin (RMP) (Sigma-Aldrich) was also used to assess the inhibition and inducibility of the CYP1A2 and CYP3A4, respectively.

Morphological analysis of bile canaliculi

During treatment with DCFA (5(6)-Carboxy-2',7'-dichlorofluorescein (Abcam), images were captured using the EVOS™ FL Auto Imaging System in the GFP channel. The resulting image dataset, together with the images obtained after immunofluorescence staining of BSEP, were processed using FIJI software to threshold the background signals. Gaussian smooth treatment and the skeleton plug-in were then used to highlight the bile canalicular network, and then Volume viewer and 3D viewer were used to generate 3D reconstructions. Analysis of the skeleton datasets, produced for all the surfaces and sections, enabled quantification of the average lengths of the bile canaliculi.

Supplemental Tables

Table S1: Protocol for hiPSC differentiation into hepatoblasts, related to figure 1

	Day	Medium	Growth factors and cytokines
Hepatoblast differentiation	0	RPMI/B27	CHIR (5 nM)
	1-4	RPMI/B27	Activin A (100 ng/ml) + LY294002 (10 nM)
	5-7	RPMI/B27	BMP4 (10 ng/ml) + FGF2 (20 ng/ml) + Activin A (50 ng/ml)
	8-9	RPMI/B27^{-met}	HGF (20 ng/ml) + FGF4 (30 ng/ml)
	10	RPMI/B27 : HCM™ (50:50)	HGF (20 ng/ml) + OSM (10 ng/ml) + Dex (0.1 nM)

Table S2: List of primer sequences used for gene expression analysis, related to figure 1 and figure S1

Gene	Primer 5' sequence 3'	Primer 3' sequence 5'
A1AT	CCA ACA GCA CCA ATA TCC ATC TTC	GTC CTC TTC CTC GGT GTC CTT G
ACTIN	GCA CTC TTC CAG CCT TCC TTC C (exon4)	CTG CTG TCA CCT TCA CCG TTC C (exon6)
AFP	TTT TGG GAC CCG AAC TTT CC	CTC CTG GTA TCC TTT AGC AAC TCT
ALB	CCT TTG GCA CAA TGA AGT GGG TAA CC	CAG CAG TCA GCC ATT CAC CAT AGG
APOA2	GGA GAA GGT CAA GAG CCC AGA G (exon 3)	AGC AAA GAG TGG GTA GGG ACA G (exon 4)
ASGR	TAG GAG CCA AGC TGG AGA AA	ACC TGC AGG CAG AAG TCA TC
CYP2B6	GAT TGA AGG CGT CTG GTT T	AGG GAG ATT GAA CAG GTG ATT
CYP3A4	GAT TTG GCT CCT CTG CTT CT	GCC TCC TGT GTA GTG AGA TTA C
CYP3A7	ATT CCA AGC TAT GTT CTT CAT CAT (exon 11)	AAT CTA CTT CCC CAG CAC TGA (exon 13)
GAPDH	CGG GAA ACT GTG GCG TGA TG (exon 1)	TGT GCT CTT GCT GGG GCT GGT G (exon8)
GATA4	CTA GAC CGT GGG TTT TGC AT	TGG GTT AAG TGC CCC TGT AG
HNF1α	AGA CTT CAC GCC ACC CAT CCT C	TTC CTC CGC CCC TTC TTG GTT G
HNF3β	AAC ACC ACT ACG CCT TCA ACC A (exon2)	GCA ACA CCG TCT CCC CAA AGT C (exon2)
HNF4α	CGG GTG TCC ATA CGC ATC CTT G (exon 7)	GAC CCT CCC AGC AGC ATC TCC T (exon8)
HTERT	TGT GCA CCA ACA TCT TGA AGC C	GCG TTC TTG GCT TTC AGG AT
LDL-R	GGG CTG GAA ATT GCG CTG GAC CGT C	TCA CAG ACG AAC TGC CGA GAG ATG C
OCT4	GTG GAG GAA GCT GAC AAC AA	CAG GTT TTC TTT CCC TAG CT
Bil-UGT	ATG ACC CGT GCC TTT ATC AC (exon4)	TCT TGG ATT TGT GGG CTT TC (exon5)

Table S3: List of primary antibodies used for immunofluorescence staining, related to figure 2

Protein	Ref.	Specifics
A1AT	DAKO A0012	rabbit polyclonal IgG
AFP	SC 8399	mouse monoclonal IgG
ALB	Sigma A6684	mouse monoclonal IgG
BSEP (ABCB11)	Sigma HPA019035	rabbit polyclonal IgG
CK-19	DAKO M0888	mouse monoclonal IgG
CK-8	SC 8020	mouse monoclonal IgG
CLDN1	SC 166338	mouse monoclonal IgG
CX26	SC 7261	goat polyclonal IgG
CX32	SC 7258	goat polyclonal IgG
CYP3A4	SC 53850	mouse monoclonal IgG
ECADH	DAKO M3612	mouse monoclonal IgG
EPCAM	SC 66020	mouse monoclonal IgG
HNF1α	SC 135939	mouse monoclonal IgG
HNF4α	SC 8987	rabbit polyclonal IgG
Ki67	Abcam ab80827	rabbit polyclonal IgG
MDR1	Fisher 4MA-26528	mouse monoclonal IgG
Phalloidin (Alexa Fluor 488)	Life Technologies A12381	
Alexa Fluor 488-conjugated	Life Technologies	

Table S4: Summary table of the hepatocyte functional features, related to figure 2-6. Table recapitulates the functional data recorded for iHep-Orgs and data from literature concerning 3D hPSC-derived hepatocytes, FHHs and PHHs. Data presented have been extracted from references selected on the similarity between the reported protocols and that of the present work. Data are shown as range of published values (when given in units allowing comparison).

		Published data					
	**iHep-Orgs**	**3D iPSC-derived Heps**		**3D FHHs**		**3D PHHs**	
	Experimental outcome	Ref.	Experimental outcome	Ref.	Experimental outcome	Ref.	Experimental outcome
**AFP secretion**	Not detected	(2–6)	Not quantified	(7)	Not quantified	(3,5,7,8)	Not quantified
**Albumin secretion**	7 µg/ml/24h/10^6	(2–6,8)	0.05-5 µg/ml/24h/10^6	(7)	25-100 µg/ml/24h/10^6	(3,5,7,8)	10-80 µg/ml/24h/10^6
**Glycogen synthesis (PAS staining)**	+	(2–4,6)	+			(3,9)	+
**Glycogenolysis (Glucose-6-Phosphatase activity)**	350 µg/ml/24h						
**Gluconeogenesis**	500 µg/ml/24h						
**Urea production**	45 µg/ml/24h/10^6	(6,8,9)	20-72 µg/24h/10^6			(8)	20 µg/ml/24h/10^6
**Production and secretion of bile acids**	76 µM/24h/10^6	(5,8)	4-25 µM/24h/10^6				
**Uptake and secretion of bile acids into the canalicular network (CDFA)**	+	(5,6,10)	+				
**Phase 0-III metabolism: ICG uptake/secretion**	+	(2,4)	+				
**CYP1A2 activity**	6 µM/ml/4h/10^6	(5)	1-15 RLU/GTC/10^6				
**CYP3A4 activity**	9 µM/ml/4h/10^6<br>65 RLU/ml/4h/10^6	(3,5,6,8–10)	≈ 50 RLU/ml/10^6	(7)	2.5 RLU/ml/per cell	(7,10)	≈ 60-80 RLU/ml/10^6
**UGT1A1 activity**	9 µM/ml/4h/10^6						
**ADH activity**	6 mU/ml/4h/10^6						

Supplemental references

1. Steichen C, Luce E, Maluenda J, Tosca L, Moreno-Gimeno I, Desterke C, et al. Messenger RNA- Versus Retrovirus-Based Induced Pluripotent Stem Cell Reprogramming Strategies: Analysis of Genomic Integrity. STEM CELLS Translational Medicine. 2014;3:686–691.

2. Akbari S, Sevinç GG, Ersoy N, Basak O, Kaplan K, Sevinç K, et al. Robust, Long-Term Culture of Endoderm-Derived Hepatic Organoids for Disease Modeling. Stem Cell Reports. 2019;13:627–641.

3. Gieseck III RL, Hannan NRF, Bort R, Hanley NA, Drake RAL, Cameron GWW, et al. Maturation of Induced Pluripotent Stem Cell Derived Hepatocytes by 3D-Culture. PLoS ONE. 2014;9:e86372.

4. Kulkeaw K, Tubsuwan A, Tongkrajang N, Whangviboonkij N. Generation of human liver organoids from pluripotent stem cell-derived hepatic endoderms. PeerJ. 2020;8:e9968.

5. Ouchi R, Togo S, Kimura M, Shinozawa T, Koido M, Koike H, et al. Modeling Steatohepatitis in Humans with Pluripotent Stem Cell-Derived Organoids. Cell Metabolism. 2019;30:374-384.e6.

6. Wang Z, Li W, Jing H, Ding M, Fu G, Yuan T, et al. Generation of hepatic spheroids using human hepatocyte-derived liver progenitor-like cells for hepatotoxicity screening. Theranostics. 2019;9:6690–6705.

7. Hu H, Gehart H, Artegiani B, LÖpez-Iglesias C, Dekkers F, Basak O, et al. Long-Term Expansion of Functional Mouse and Human Hepatocytes as 3D Organoids. Cell. 2018;175:1591-1606.e19.

8. Mun SJ, Ryu J-S, Lee M-O, Son YS, Oh SJ, Cho H-S, et al. Generation of expandable human pluripotent stem cell-derived hepatocyte-like liver organoids. Journal of Hepatology. 2019;71:970–985.

9. Pettinato G, Lehoux S, Ramanathan R, Salem MM, He L-X, Muse O, et al. Generation of fully functional hepatocyte-like organoids from human induced pluripotent stem cells mixed with Endothelial Cells. Sci Rep. 2019;9:8920.

10. Ramli MNB, Lim YS, Koe CT, Demircioglu D, Tng W, Gonzales KAU, et al. Human Pluripotent Stem Cell-Derived Organoids as Models of Liver Disease. Gastroenterology. 2020;159:1471-1486.e12.

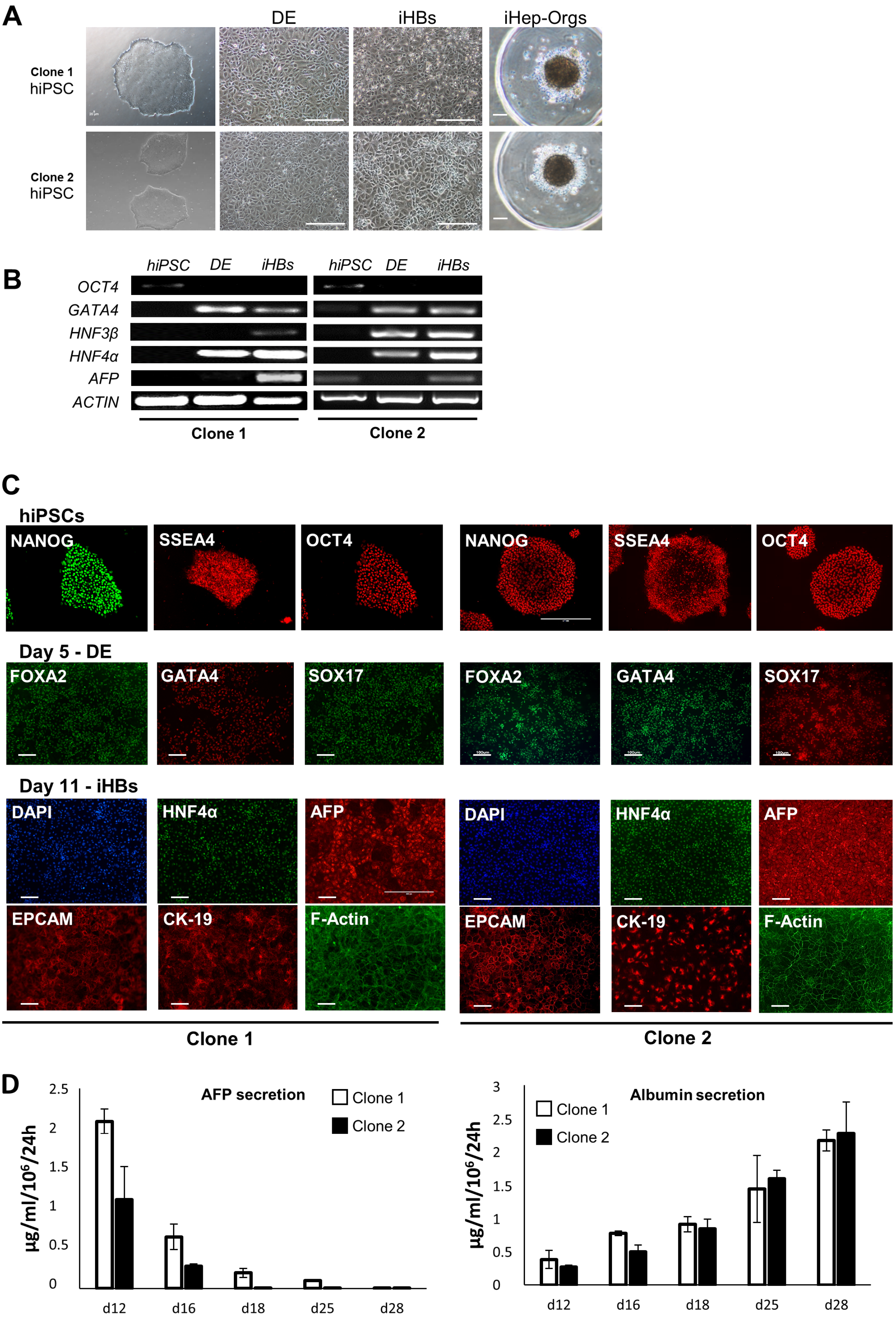
A
DE
iHBs
iHep-Orgs
Clone 1
hiPSC
Clone 2
hiPSC
B
hiPSC
DE
iHBs
hiPSC
DE
iHBs
OCT4
GATA4
HNF3β
HNF4α
AFP
ACTIN
Clone 1
Clone 2
C
hiPSCs
NANOG
SSEA4
OCT4
NANOG
SSEA4
OCT4
Day 5 - DE
FOXA2
GATA4
SOX17
FOXA2
GATA4
SOX17
Day 11 - iHBs
DAPI
HNF4α
AFP
DAPI
HNF4α
AFP
EPCAM
CK-19
F-Actin
EPCAM
CK-19
F-Actin
Clone 1
Clone 2
D
AFP secretion
Clone 1
Clone 2
µg/ml/10⁶/24h
2.5
2
1.5
1
0.5
0
d12
d16
d18
d25
d28
Albumin secretion
Clone 1
Clone 2
µg/ml/10⁶/24h
3
2.5
2
1.5
1
0.5
0
d12
d16
d18
d25
d28

Figure S1. Assessment of hiPSC differentiation into iHBs, related to figure 1.
(A) Brightfield images of hiPSC clone 1 (upper panel) and clone 2 (left panel). From left to right: hiPSC colonies before differentiation, at the definitive endoderm stage and at hepatoblastic stage. Scale bars for all brightfield images = 100 µm.
(B) RT-PCR analysis of the gene expression of stemness and hepatoblastic markers over time for the hiPSCs, the definitive endoderm (DE) and iHBs. Clone 1 (left panel) and clone 2 (right panel).
(C) Immunofluorescence staining of stemness, endoderm and hepatoblast markers from day 0 to day 11 of the differentiation protocol. Clone 1 (left panel) and clone 2 (right panel). Scale bar = 500 µm for day 0 - hiPSCs. Scale bars = 100 µm for day 5 – DE and day 11 – iHBs.
(D) ELISA quantification of AFP and ALB secretion of clone 1 (white bars) and clone 2 iHep-Orgs (black bars). Histograms represent mean ± SD (n=16).

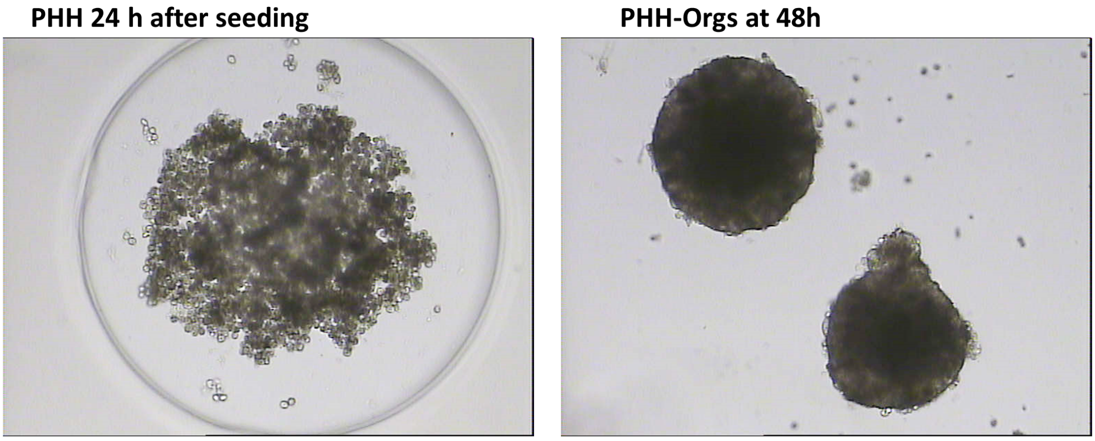

Figure S2. PHH self-assembling into organoids in agarose µwells, related to figure 1. Left panel: PHHs 24h after seeding into agarose µwells. Right panel: PHH-Orgs formed after 48h of culture

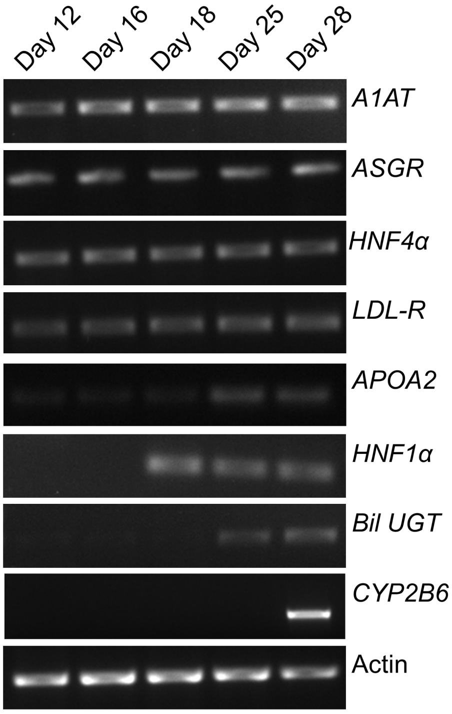

Figure S3. Gene expression of hepatocyte markers, related to figure 1. RT-PCR analysis of hepatic gene expression in iHep-Orgs over time.

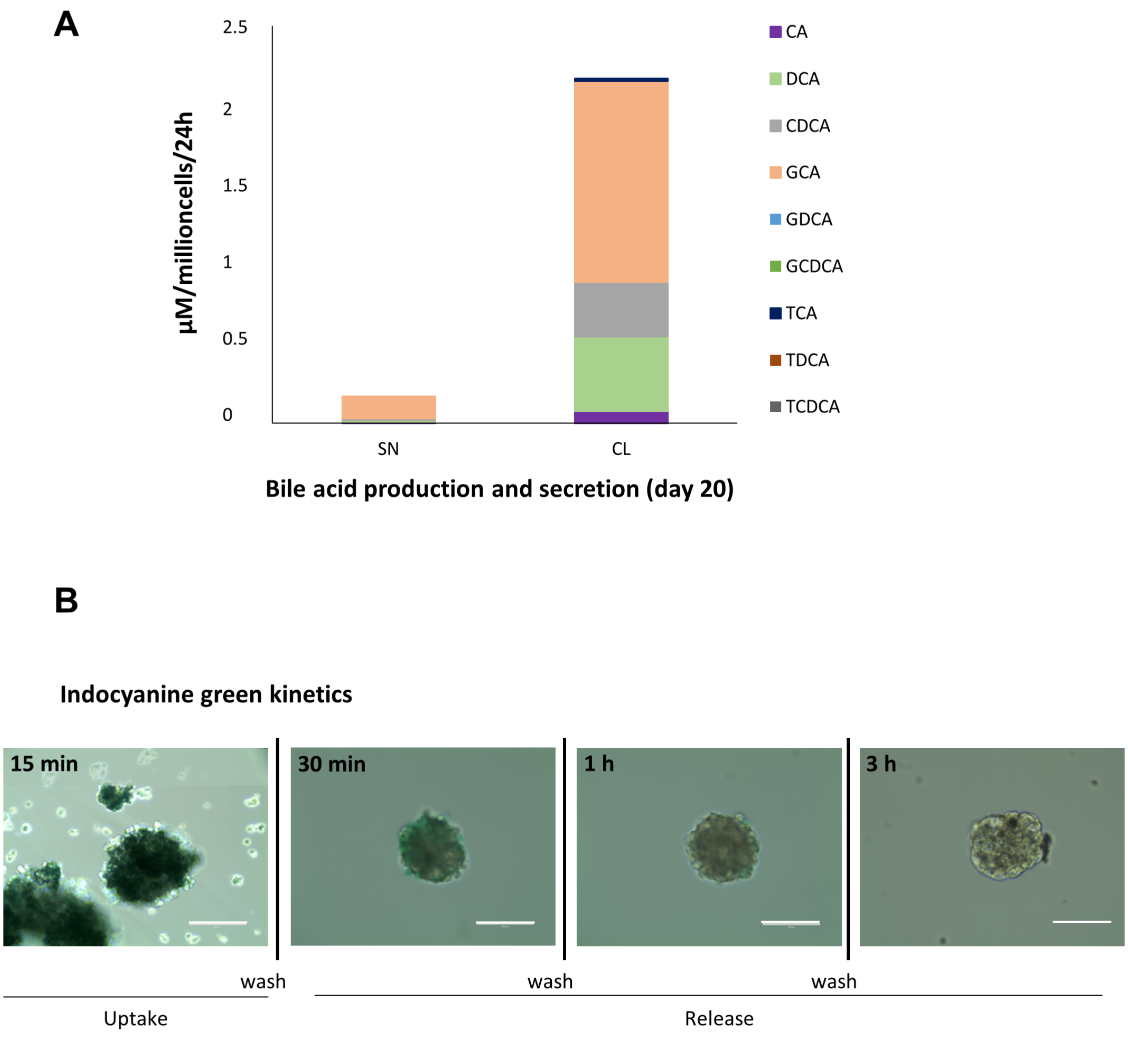

Figure S4. Assessment of iHep-Org functions associated with polarization, related to figure 3. (A) Quantification of bile acid (BA) production in cell lysates and secretion into culture supernatants from iHep-Orgs at day 20 of culture. SN = supernatant; CL= cell lysate. The higher values in BA concentration detected in the CL compared to the SN could be due to i) the quantities excreted are insufficient to be quantified (excessive dilution) ii) the incomplete maturation of the bile canalicular network in iHep-Orgs at day 20.

(B) Indocyanine Green (ICG) uptake and release assay in iHep-Orgs. Scale bar = 200 µm. The assay allows the assessment of OATP1B3, NTCP and MRP2 functions associated with polarization and transport.
